# Meteorological Table

**Published:** 1813-12

**Authors:** 


### METEOROLOGICAL TABLE.

*From October 25, to November 25,* 1813.

| | D. | Therm. | | | Barom. | | Hygrom. *Dry.——Damp.* | | | Winds. | Atmos. Variation: |
|---|---|---|---|---|---|---|---|---|---|---|---|
| | 26 | 43 | 45 | 43 | 30 | — | 10 | — | — | E. | F.. —.. C.. —... |
| | 27 | 43 | 43 | 42 | $29^{9}$ | $^{8}$ | 8 | 6 | 11 | NE.. | R. —. C. |
| | 28 | 42 | 44 | 41 | — | $29^{9}$ | 11 | 13 | 10 | N.NE. | C. R.. |
| | 29 | 39 | 44 | 40 | $29^{6}$ | — | 11 | — | — | NE. | F. R. C... |
| | 30 | 37 | 50 | 51 | $29^{8}$ | $29^{3}$ | 12 | 19 | 22 | W. | Fog. R.. —. |
| | 31 | 50 | 48 | 44 | $29^{1}$ | $29^{2}$ | 24 | 20 | 19 | W.. | F.. —.. Fog.. |
| 🌗 | 1 | 40 | 47 | 42 | $29^{8}$ | $^{6}$ | 20 | 15 | 16 | W. | F. —.. —. |
| | 2 | 40 | 49 | 46 | $29^{7}$ | $^{5}$ | 19 | 20 | 21 | W.. | F.. R... R. |
| | 3 | 41 | 45 | 43 | 30 | $30^{3}$ | 15 | 10 | 16 | N.. | F.. —.. —.. |
| | 4 | 36 | 46 | 42 | — | — | 15 | 15 | 15 | NW.. | F. —... —.. |
| | 5 | 39 | 47 | 45 | — | $30^{2}$ | 16 | 18 | 20 | N. | Fog... F... —... |
| | 6 | 39 | 46 | 42 | 30 | $29^{8}$ | 16 | 15 | 12 | W. | F... —... —... |
| | 7 | 43 | 49 | 47 | $29^{6}$ | $^{5}$ | 10 | 16 | 19 | S. | R.. C. R.. |
| ○ | 8 | 46 | 49 | 48 | — | — | 20 | — | — | W.. | F.. R... F... |
| | 9 | 49 | 53 | 48 | $29^{6}$ | $^{7}$ | 19 | 15 | 17 | W.. | F.. —.. —... |
| | 10 | 49 | 52 | 50 | — | $^{6}$ | 20 | — | 22 | W.. | C.. R.. C.. |
| | 11 | 50 | 55 | 52 | — | $^{7}$ | 20 | — | 16 | NW.. | F.. —... —.. |
| | 12 | 51 | 54 | 45 | $^{8}$ | — | 15 | — | 16 | NW... | C.. —... C. |
| | 13 | 41 | 45 | 41 | — | — | — | — | 17 | NW.. | C. C.. —... |
| | 14 | 38 | 42 | 44 | $29^{5}$ | $^{4}$ | 15 | — | — | W. | F.. —.... R.. |
| 🌓 | 15 | 40 | 45 | 41 | $^{3}$ | — | 14 | 12 | 16 | NE. | C.. —... |
| | 16 | 39 | 42 | 43 | $^{2}$ | — | — | — | — | NE.. | F. —... R.. |
| | 17 | 38 | 40 | 37 | $29^{3}$ | — | 15 | — | — | W. | C.. Snow... C.. |
| | 18 | 38 | 41 | 39 | $29^{5}$ | $^{7}$ | — | — | — | W.. | C.. F.. —.. |
| | 19 | 42 | 47 | 50 | — | — | 16 | 20 | 22 | W. | R.. —. C.. |
| | 20 | 50 | 54 | 52 | $29^{8}$ | $^{9}$ | 22 | — | — | W.. | C.. —.. —... |
| | 21 | 51 | 56 | 52 | — | — | 20 | — | 17 | SW. | C... |
| ● | 22 | 52 | 55 | 50 | 30 | — | 14 | 15 | — | S. | C... |
| | 23 | 49 | 51 | 47 | 30 | — | 15 | — | 12 | S. | C... |
| | 24 | 47 | 49 | 44 | 30 | $30^{1}$ | 12 | — | — | E. | C... |
| | 25 | 44 | 47 | 42 | — | — | — | — | 11 | E. | C... |

Quantity of rain from the 26th of October to the 25th of November, two inches $\frac{25}{100}$.

On the 8th, storm of thunder, with heavy rain, and on the 17th, first fall of snow this winter. Cases of chlorea have still occurred, but the prevailing disease has been complaints of the chest, generally affecting the mucous membrane, but sometimes the parenchemia of the lungs, especially in children.

